# Immunization with the Recombinant Cholera Toxin B Fused to Fimbria 2 Protein Protects against *Bordetella pertussis* Infection

**DOI:** 10.1155/2014/421486

**Published:** 2014-05-13

**Authors:** Noelia Olivera, Celina E. Castuma, Daniela Hozbor, María E. Gaillard, Martín Rumbo, Ricardo M. Gómez

**Affiliations:** ^1^Instituto de Biotecnología y Biología Molecular, Facultad de Ciencias Exactas, Universidad Nacional de La Plata, CCT-La Plata, CONICET-UNLP, Calle 49 y 115, 1900 La Plata, Argentina; ^2^LISIN, Facultad de Ciencias Exactas, Universidad Nacional de La Plata, Calle 48 y 115, 1900 La Plata, Argentina

## Abstract

This study examined the immunogenic properties of the fusion protein fimbria 2 of *Bordetella pertussis* (Fim2)—cholera toxin B subunit (CTB) in the intranasal murine model of infection. To this end *B. pertussis* Fim2 coding sequence was cloned downstream of the cholera toxin B subunit coding sequence. The expression and assembly of the fusion protein into pentameric structures (CTB-Fim2) were evaluated by SDS-PAGE and monosialotetrahexosylgaglioside (GM1-ganglioside) enzyme-linked immunosorbent assay (ELISA). To evaluate the protective capacity of CTB-Fim2, an intraperitoneal or intranasal mouse immunization schedule was performed with 50 *μ*g of CTB-Fim2. Recombinant (rFim2) or purified (BpFim2) Fim2, CTB, and phosphate-buffered saline (PBS) were used as controls. The results showed that mice immunized with BpFim2 or CTB-Fim2 intraperitoneally or intranasally presented a significant reduction in bacterial lung counts compared to control groups (*P* < 0.01 or *P* < 0.001* ,* resp.). Moreover, intranasal immunization with CTB-Fim2 induced significant levels of Fim2-specific IgG in serum and bronchoalveolar lavage (BAL) and Fim2-specific IgA in BAL. Analysis of IgG isotypes and cytokines mRNA levels showed that CTB-Fim2 results in a mixed Th1/Th2 (T-helper) response. The data presented here provide support for CTB-Fim2 as a promising recombinant antigen against *Bordetella pertussis* infection.

## 1. Introduction

Pertussis or whooping cough is an acute respiratory disease whose principal etiological agent is the gram-negative bacterium* Bordetella pertussis* [1]. The clinical manifestations are more severe in infants than in adolescents or adults, who are now recognized as the main source of infection [2]. The best way to prevent pertussis is vaccination with either whole cellular (wP) or acellular (aP) vaccines [3]. Protective immunity generated by wP appears to be mediated largely by Th1 cells, whereas less efficacious alum-adjuvanted aP induce strong antibody Th2 responses [4].

Despite widespread pertussis immunization in childhood for more than 50 years, pertussis is considered to be the most poorly controlled bacterial vaccine-preventable disease [5] and remains an endemic disease with regular epidemics [6]. Currently, there are an estimated 16 million cases and 195,000 deaths due to pertussis globally each year, most of them in developing countries [1]. The most vulnerable to the disease correspond to groups of unvaccinated infants, partially vaccinated children, and persons who have completed the immunization schedule with waning immunity [1]. In addition, since the early 1980s there has been an increase in reported cases of pertussis [5], even in countries with a high vaccination coverage rate [7]. Waning immunity conferred by vaccines, increased recognition, changes in diagnostic testing and reporting, and adaptation of the agent to immunity induced by vaccines are some of the factors that may have contributed to this increase [5].

Taken together, it is clear that additional vaccine approaches are needed. Some of the new approaches under trial include vaccination of newborns and additional booster doses for older adolescents and adults. Innovative vaccines are also being studied [1]. In this regard, since infection by* B. pertussis* is usually restricted to the airways, an interesting alternative may be mucosal vaccination [8, 9]. It has been shown that mucosal vaccination is the best way to achieve a strong cellular and humoral immune response in airways as well as systemically [10]. There are also important logistic reasons that have made mucosal immunization attractive for public health use. Mucosal vaccines should be easier and cheaper to administer than parenteral vaccines and also have a lower risk of transmitting hepatitis B virus and HIV infections [11].

However, most protein antigens are poorly immunogenic and potent adjuvants are often needed to enhance immunity [12]. The cholera toxin B subunit (CTB) is among the most potent mucosal adjuvants known [13, 14]. CTB is the pentameric nontoxic portion of cholera toxin (CT) responsible for the binding of the holotoxin to the monosialotetrahexosylgaglioside (GM1 ganglioside) receptor [15]. Chemical and genetic conjugations of CTB with different heterologous antigens from simian immunodeficiency virus and* Schistosoma mansoni*, among others, have shown promising results [16, 17].

The fimbriae (Fim) proteins are promising as immunogens to be used by the mucosal route because they act as pathogen-associated molecular patterns (PAMPs) [18, 19] and, in the case of Fim2 and 3 of* B. pertussis,* have known immunogenic properties and although Fim3 seems to exhibit lower protective capacity than Fim2 when isolated from* B. pertussis,* both have justified their presence in most recent acellular vaccines [20, 21].

In this study, we constructed a histidine-tagged CTB-Fim2 fusion protein in order to evaluate its protective capacity and immunogenic properties in a* B. pertussis* respiratory infection murine model. The results presented here showed that CTB-Fim2 is a promising antigen against* B. pertussis *infection.

## 2. Materials and Methods

### 2.1. Strain and Growth Conditions

The* Escherichia coli* strain DH5*α* (Invitrogen, USA) was used for all routine cloning experiments, whereas the BL21(SI) and BL21Star (DE3) *E. coli* competent cells (Invitrogen) were used for recombinant protein expression. The* B. pertussis *strain used in this study was Tohama phase I [22–24] obtained from the Pasteur Institute, Paris, France. The strain from glycerol stock was cultured on Bordet-Gengou agar (BGA, Difco) supplemented with 1% glycerol and 10% (v/v) defibrinated sheep blood and incubated at 36°C for 3 days. The bacteria was then replated in the same medium for 24 h and the subcultures were grown in Stainer-Scholte liquid medium (SS) [25], with shaking at 36°C for 20 h.

### 2.2. Cloning of Recombinant Proteins

The* fim*2 (621 bp) gene, from* B. pertussis* strain Tohama phase I, was amplified from genomic DNA by PCR. The mixture was subjected to a program consisting of a DNA denaturation step at 94°C for 2 min, 35 cycles at 94°C for 15 s, 48°C for 15 s, and 72°C for 40 s. The following oligonucleotides were used for cloning into pET-TOPO 200 and pAEctxB plasmids, respectively: Fim2F 5′CACCATGCCATTGATCTCG3′ and Fim2R 5′TTCGCTCCTGCATGGAATAC3′; CTBFim2F 5′TGGTTC**ACGCGT**ATGTTACCCATGCAAATCCC3′ and CTBFim2R 5′CTGAT**AAGCTT**CTAGGGGTAGACCACGG3′. In bold are the* Mlu*I and* Hind*III restriction sites, respectively. The amplified products were cloned into pET-TOPO 200 vector and transferred to pDEST17 according to the manufacturer's instructions (Invitrogen) or into pAE-ctxB [26] using the* Mlu*I and* Hind*III sites in order to generate the pDEST17-*fim2* and pAE-*ctxB*-*fim2* plasmids. The recombinant clones were confirmed by PCR and sequenced.

### 2.3. Expression and Purification of the Recombinant Proteins

The expression and purification of rFIM2 and CTB-Fim2 was performed as previously described for other recombinant proteins [27, 28]. Briefly, BL21(SI)* E. coli *competent cells were transformed with the pDEST17-*fim2* or pAE-*ctxB*-*fim2* plasmids and grown overnight (ON) at 37°C. Ampicillin-resistant colonies were inoculated in 5 mL on Luria Bertani (LB) medium with ampicillin (50 *μ*g/mL) without NaCl and grown ON at 37°C. On the following day, cultures were diluted 50-fold in LB-amp without NaCl and grown until A_600_ reached 0.8, when NaCl was added to the medium at a final concentration of 300 mM. After 3 h, cells were collected by centrifugation, resuspended in 10 mL lysis buffer, pH 8.0 (Tris-Cl 20 mM and NaCl 100 mM), and lysed by sonication. Cellular lysates were centrifuged at 14,000 rpm for 30 min. Both recombinant proteins were recovered from the inclusion bodies and solubilized with 10 mL of solubilization buffer (8 M urea, 50 mM Tris-Cl, 500 mM NaCl, pH 8.0). The material was then dialyzed against 2 L of refolding buffer (500 mM NaCl, 50 mM Tris-Cl, pH 8.0). Refolded protein solution was adsorbed to the Ni^2+^-NTA resin (Invitrogen) and washed with ten volumes of binding buffer (100 mM NaCl, 20 mM Tris-Cl, pH 8.0) containing 5, 20, 40, and 60 mM imidazole. The proteins were eluted with five volumes of the same solution containing 250 mM imidazole. Fractions were analyzed by 10% SDS-PAGE. The purified proteins were dialyzed in one step. The equilibrium was established using 2 L of a 10 mM Tris-Cl, pH 8.0, 20 mM NaCl, and 0.1% (m/v) glycine solution. The identity of the expressed protein and its molecular weight were confirmed by MALDI-TOF mass spectrometry. Searches for and the identification of peptides were performed using a licensed version of MASCOT software (Matrix Science) with a peptide tolerance of 50 ppm. Monoisotopic peptide masses were used to search the database, allowing a molecular mass range for 2-DE analyses of 15%. Purified CTB was purchased from Sigma (Argentina). For immunogenicity studies, contaminant LPS was removed from aliquots of recombinant proteins using the Detoxi-Gel Endotoxin Removing Gel columns (Thermo Scientific-Pierce, USA), according to the manufacturer's instructions. The purification of BpFim2 was similar to a procedure described elsewhere [29].

### 2.4. Limulus Amebocyte Lysate Assay (LAL Test)

The chromogenic LAL assay for endotoxin activity of the protein samples was performed using the QCL-1000 kit (Bio-Whittaker, MD), according to the manufacturer's instructions. In all cases, the final LPS content was less than 5 × 10^−6^ *μ*g/mL.

### 2.5. Immunoblotting

The immunoblot analysis (IB) was performed as previously described [28]. Briefly, aliquots were subjected to SDS-PAGE and then transferred to nitrocellulose membranes (GE Healthcare). Membranes were blocked with 5% nonfat dried milk in PBS containing 0.05% Tween 20 (PBS-T) and then incubated with a mouse antiserum (1 : 2500) against CTB in 5% nonfat dried milk/PBS-T for 2 h at RT. After washing, the membrane was incubated with HRP-conjugated anti-mouse IgG (1 : 5000; Sigma) in 5% nonfat dried milk/PBS-T for 1 h. The bands were revealed with ECL reagent kit chemiluminescence substrate (GE Healthcare).

### 2.6. Pentamer Analysis Assays

To determine whether CTB-Fim2 folds into pentamers during SDS-PAGE, samples were subjected to denaturing and nondenaturing conditions. In the latter case, samples were not boiled, and the sample buffer used did not contain *β*-mercaptoethanol. The ability of the CTB pentamers to bind to their cellular receptor was assessed using a GM1-ELISA assay. This protocol was adapted from a previously published study [28]. Briefly, 96-well plates (Nunc) were coated with 0.5 *μ*g per well (100 *μ*L) of GM1 (Sigma) in PBS, pH 7.4 at 4°C for 16 h. As a negative control, the wells were coated with bovine serum albumin (BSA) at the same concentration (5 *μ*g/mL) in PBS pH 7.4. Commercial CTB was used as a positive control. Plates were blocked and then washed five times. Afterwards, the wells were incubated with 100 *μ*L of the recombinant protein serially diluted in PBS 1/2 from ~300 nM to 146 pM for 2 h at 37°C and then washed. Microtiter plates were incubated for 1 h at 37°C with a 1/2500 dilution of a polyclonal anti-CTB serum [28] in 5% nonfat dried milk/PBS-T and washed. Thereafter, they were incubated with the anti-mouse conjugated to HRP (Santa Cruz Biotechnology, USA) diluted 1/4000 for 1 h at 37°C. After washing, plates were revealed by the addition of 10 mg o-phenylenediamine (OPD) in 10 mL of a 0.2 M citrate-phosphate buffer, pH 5.0, in the presence of 10 *μ*L of H_2_O_2_. The reaction was stopped by the addition of 4 M H_2_SO_4._ The absorbance was measured at 492 nm in all cases.

### 2.7. Immunization of Mice and Sample Collection

Groups of 4–8 and 3-4 week-old female BALB/c mice (Instituto Biológico Argentino, Argentina) received 50 *μ*g of purified CTB-Fim2 intraperitoneal or intranasally in 50 *μ*L of PBS on days 0, 12, and 24 with aluminum hydroxide as an adjuvant (0.2 mg/mL, IP) or without external adjuvant (IN). Mice immunized with CTB (5 *μ*g, IN only), rFim2, or BpFim2 alone (50 *μ*g) and mice inoculated with 50 *μ*L of PBS were used as control groups.

Blood samples were obtained from a facial vein at days 12, 24, and 36, and the obtained sera were stored at −20°C. BAL fluid was collected postmortem after the challenge by flushing the lungs twice with PBS. An incision was made in the neck, so as to expose the trachea. Using a 5 mL syringe, 1 mL of PBS was slowly introduced into the lungs via the trachea and then syringed out. This was repeated twice. The protease inhibitor phenylmethylsulfonyl fluoride (Sigma) was added to each sample at a concentration of 1 mM following collection, and samples were kept at −20°C until use. At least two independent experiments were performed with consistent results.

### 2.8. Antibody Determination by ELISA

Ninety-six well plates were coated with 5 *μ*g mL^−1^ of rFim2/well in carbonate-bicarbonate buffer, pH 9.6 at 4°C ON. The plates were washed three times with PBS-T. Nonspecific binding was reduced by blocking the plates with 300 *μ*L/well of 5% nonfat milk/PBS-T (m/V) at 37°C for 1 h. The plates were washed three times with PBS-T. Samples that were to be tested for Fim2-specific antibodies were diluted 1/20 for sera IgG, IgG1, IgG2a, and IgA (the mucosal fluids were not previously diluted) in PBS-T, with 50 *μ*L being added to the plate in duplicate and serially diluted 2-fold down the plate. A final 1/64 dilution was used for comparison and graphical purposes. The plates were incubated at 37°C for 1 h and then washed three times with PBS-T. Bound antibodies were detected using at appropriate dilutions commercial peroxidase conjugated anti-mouse, IgG, IgG1a, IgG2, and IgA (Santa Cruz Biotechnology). The subsequent steps were performed as described in GM1-ELISA.

### 2.9. Protection Assay in a Mouse Respiratory Model

The challenge assay was performed using the mouse respiratory model of* B. pertussis* infection as described previously [30]. Briefly, cohorts of BALB/c mice were immunized as described above, and 12 days after the last dose of immunization, they were challenged with a suspension of 5 × 10^7^ CFU of virulent* B. pertussis* in 50 *μ*L of PBS. Inoculation was performed by pipetting the inoculum into the nostrils. Animals were euthanized by CO_2_ overdose 8 days postinfection (PI), and the lungs were excised. Left lung was used for bacterial recovery as described previously [30], and the right lung was split in two samples, one used for cytokine level and the other for routine histological examination.

All animal experiments were performed according to our Institute's Ethical Committee and the National Institutes of Health guidelines [31].

### 2.10. RNA Isolation and RT-PCR

Total RNA was isolated from lung tissue by mechanical homogenization in Trizol reagent (Invitrogen, Argentina), as recommended by the manufacturer. DNase treatment was performed with an RNase-free DNase Kit (Qiagen, Germany). cDNA was synthesized from 500 ng of total RNA with 15 mM of random hexamers and SuperScript III reverse transcriptase (Invitrogen, Argentina), according to the manufacturer's instructions.

### 2.11. Real-Time PCR

All q-PCR studies were performed with a Line-Gene instrument (Bioer Technology, China). The TAQurate green real-time PCR MasterMix (Epicentre Biotechnologies) was used for all reactions, following the manufacturer's instructions. Initial denaturation was carried out at 94°C for 10 min, followed by 65 cycles of 20 s at 94°C, 15 s at the respective annealing temperatures and 15 s at 72°C each, and a final extension at 72°C for 2 min. A melting curve analysis was performed immediately after amplification at a linear temperature transition rate of 0.3°C/s from 70°C to 89°C with continuous fluorescence acquisition. The size of all PCR products was confirmed by agarose gel electrophoresis. Transcription levels of IL-4 (F 5′CATCGGCATTTTGAACGAGGTCA3′; R 5′CTTATCGATGAATCCAGGCATCG3′); and IFN-*γ* (F 5′CTTGGATATCTGGAGGAACTGGC3′; R 5′GCGCTGACCTGTGGGTTGTTGA3′) were measured in all samples and normalized to *β*-actin levels (F 5′GCTTCTTTGCAGCTCCTTCGT3′; R 5′CATTCATGTTTCGAATCATTTCAAA3′).

### 2.12. Statistical Analysis

A one-way ANOVA test was used to determine significant differences in the assays, and data are expressed as mean ± SEM. When necessary, data were analyzed by one-way analysis of variance followed by the Bonferroni multiple comparison test to determine significant differences between groups. *P* < 0.05 was considered statistically significant.

## 3. Results

### 3.1. Expression and Purification of CTB-Fim2

**Figure 1:**
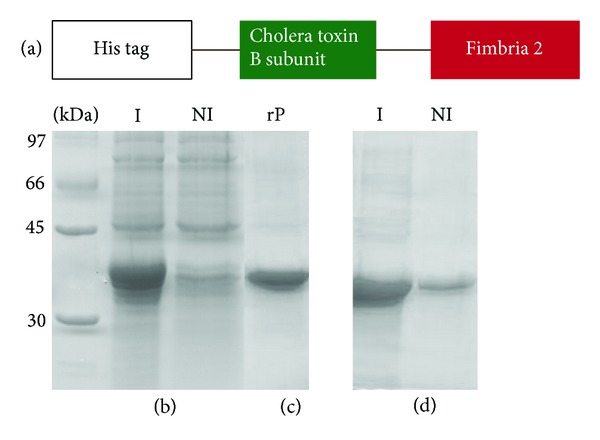
Immunogen design, expression, purification, and immunoblot analysis of the CTB-Fim2 fusion protein. (a) Schematic representation of immunogen design. (b) Protein expression and purification in the* E. coli *(SI) strain transformed with pAE-CTB-Fim2. Protein expression was assayed using a 10% SDS-PAGE gel and shows a 36 kDa band. (c) Analysis of the recombinant CTB-Fim2 protein after elution from Ni^2+^-charged beads with 250 mM imidazole. (d) Reactive protein bands probed with a mouse antiserum (1 : 2500) against CTB. NI and I stand for noninduced and induced bacterial extract, respectively.

The design used to construct the recombinant plasmid is illustrated in Figure 1(a). The fusion protein was expressed and purified as described previously [28]. The results are shown in Figures 1(b) and 1(c), respectively. As expected, the molecular weight of CTB-Fim2 was 36 kDa. The presence of CTB in the fusion protein was confirmed by immunoblotting using a murine anti-CTB antiserum (Figure 1(d)) and by MALDI-TOF mass spectrometry. While a positive result was detected for CTB-Fim2, no band was detected in the nontransformed* E. coli* extract or when the primary antibody was omitted (data not shown). These data demonstrated that the recombinant* E. coli* harboring the plasmid express the recombinant protein recognizable by anti-CTB antibodies.

### 3.2. Pentamer Conformation of CTB-Fim2

**Figure 2:**
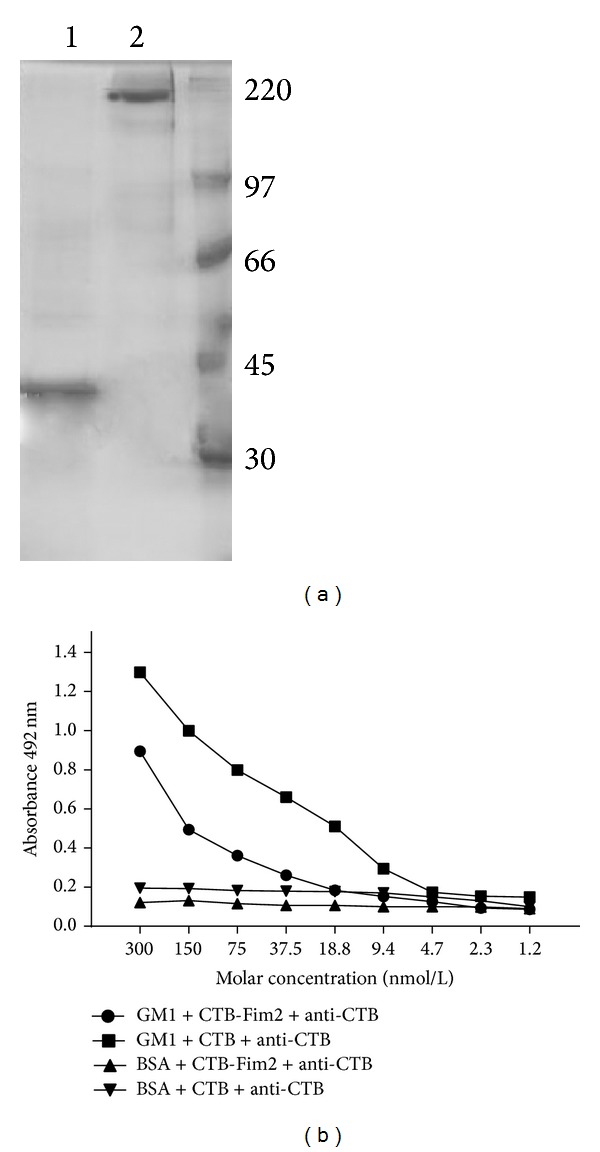
Pentamer formation of CTB-Fim2. (a) SDS-PAGE with purified CTB-Fim2. Lane 1 is the sample boiled in reducing conditions while lane 2 is the same protein sample not subjected to boiling and in nonreducing conditions. Bands of 36 and 180 kDa of CTB-Fim2 are shown, respectively. (b) GM1-ELISA to verify the ability of the pentamer to bind GM1-ganglioside using recombinant CTB-Fim2. Commercial CTB was used as a positive control. The 96-well plates were coated with GM1 or BSA and mouse anti-CTB antisera (1 : 2500).

The oligomeric structure of CTB-Fim2 was analyzed by gel electrophoresis.  Figure 2(a) (lane 1) shows that CTB-Fim2, previously solubilized in denaturing Laemmli sample buffer and boiled, migrated with a MW of 36 kDa. In contrast, when protein was solubilized in nondenaturing Laemmli sample buffer without boiling (lane 2) it migrated with a MW of 180 kDa. These results suggest that native CTB has a pentameric structure after the refolding process.

The pentameric conformation was also evaluated by its ability to bind to its receptor GM1-ganglioside in an ELISA assay since monomeric CTB has a low affinity for GM1 [32]. For detection, anti-CTB serum was used (Figure 2(b)). The results showed that commercial CTB and purified CTB-Fim2 were similarly able to bind GM1 in a concentration-specific manner. These results strongly suggest that CTB-Fim2 has a pentameric structure and indicate that the presence of Fim2 does not abrogate either the formation of the pentamer or binding to its GM1 receptor.

### 3.3. Protective Capacity of CTB-Fim2

To evaluate the protective capacity against* B. pertussis* infection of CTB-Fim2 immunization, we employed a previously described mice model [30]. Balb/c mice were immunized intraperitoneally or intranasally three times with (50 *μ*g/dose) the recombinant CTB-Fim2, rFim2, and BpFim2 and were challenged with a 5 · 10^7^ CFU of virulent* B. pertussis* Tohama phase I strain/50 *μ*L suspension in PBS 12 days following the last dose. Mice treated with CTB (5 *μ*g, IN only) or 50 *μ*L of PBS were used as negative controls.

**Figure 3:**
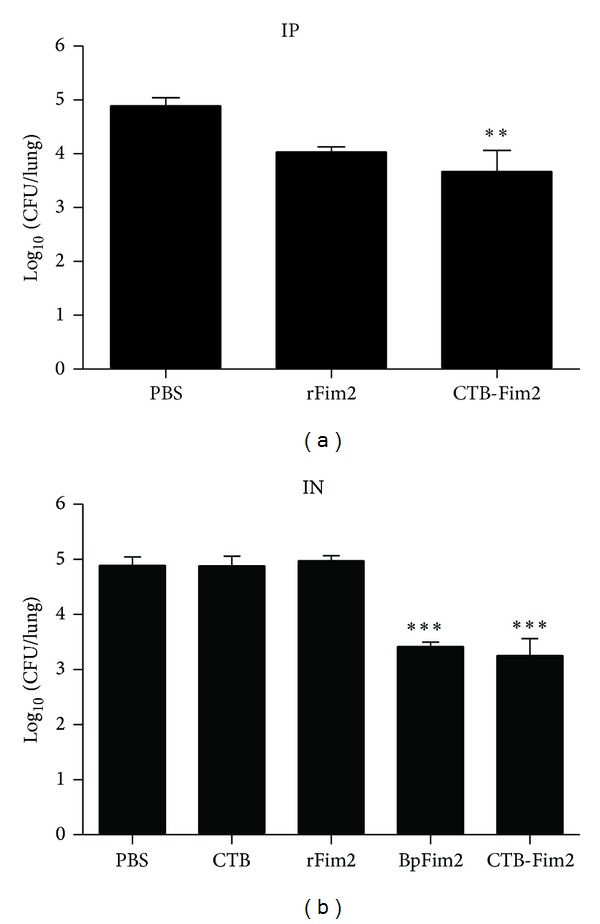
Protective capacity of CTB-Fim2. Groups of animals were immunized (a) intraperitoneally or (b) intranasally three times with PBS, CTB (IN only), rFim2, BpFim2, or CTB-Fim2 every 12 days followed by IN challenge with 5 × 10^7^ UFC of the Tohama I strain of* B. pertussis*. At 8 days postinfection, animals were killed and lung samples were collected for UFC counting. Each point represents the mean (± standard error) of 8 animals (**P < 0.001 versus PBS; ***P < 0.0001 versus PBS, CTB, and rFim2).

The results obtained at 8 days after the challenge showed a significant reduction in bacterial counts in mice immunized either by the intraperitoneal or the intranasal route with CTB-Fim2 compared to the controls treated with PBS or CTB (IP 3.7 ± 0.2 versus 5.3 ± 0.2 logs CFU/lung, *P* < 0.01) or (IN 3.2 ± 0.2 versus 4.9 ± 0.1 logs CFU/lung, *P* < 0.001), respectively. In contrast, intraperitoneal or intranasal immunization with rFim2 did not significantly reduce the bacterial counts in lungs compared to control groups (4.8 ± 0.1 and 5.3 ± 0.1 logs CFU/lung, resp.). As expected, a significant decrease was seen between BpFim2, isolated from* B. pertussis* and used as a positive control, and control groups either by the intraperitoneal or the intranasal route (*P* < 0.001) (Figure 3).

There were no statistically significant differences between mice immunized intraperitoneally or intranasally with CTB-Fim2 (3.7 ± 0.2 versus 3.2 ± 0.2 CFU/lung).

These results indicate that intraperitoneal or intranasal administration of CTB-Fim2 was effective in protecting mice against intranasal challenge with* B. pertussis* with a dose of 50 *μ*g of immunogen. Moreover, CTB improved the protective capacity of rFim2 when it was genetically fused to the antigen.

### 3.4. Serum IgG Levels Following Immunization

**Figure 4:**
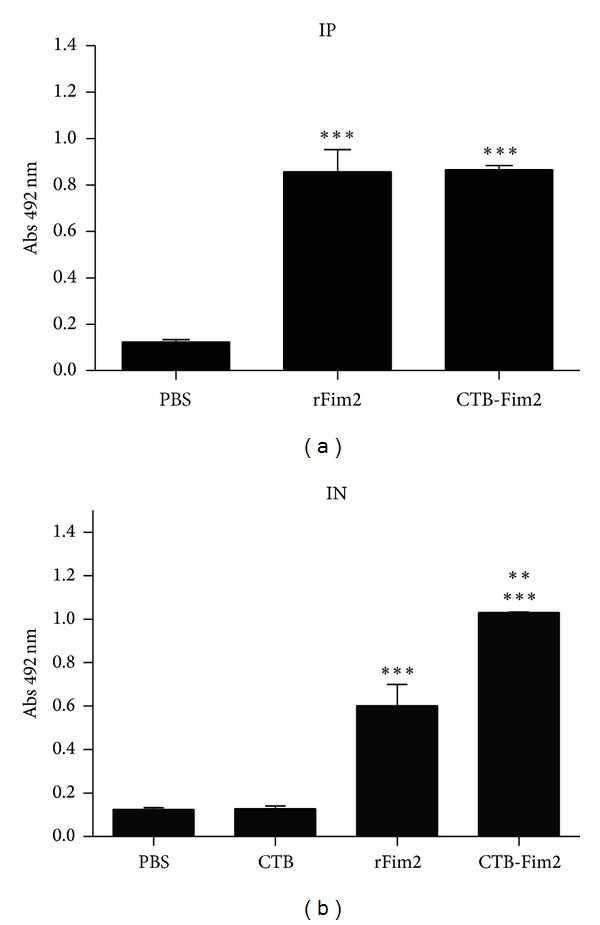
Specific IgG antibodies in serum. Induction of serum anti-Fim2 IgG after intraperitoneal (a) or intranasal (b) immunization with PBS, CTB, rFim2, and CTB-Fim2. Anti-Fim2 IgG titers of individual serum collected immediately before challenge on day 36 are shown. Values represent the OD at 492 nm and show the mean (±standard error) of 8 animals (**P < 0.001 versus rFim2 IN; ***P < 0.0001 versus PBS and CTB IN and IP).

To further characterize the immunogenic properties of CTB-Fim2, we study the humoral response induced in serum samples collected during the intraperitoneal or intranasal immunization protocols, by ELISA. As expected, the specific humoral response increased after each immunization in all groups. To simplify the analysis, only the values of serum IgG at day 35, one day before challenge with* B. pertussis,* are shown (Figure 4). Either intraperitoneal or intranasal immunization with rFim2 or CTB-Fim2 generated a specific serum IgG response (DO at 492 nm = 0.9 and 0.6, resp., for rFim2; 0.9 or 1.2 for CTB-Fim2, resp.; *P* < 0.01 and 0.001, resp.) (see Figure 4). These results show that meanwhile rFim2 and CTB-Fim2 generated similar levels of anti-Fim2 serum IgG after intraperitoneal immunization, the level of anti-Fim2 serum IgG elicited by CTB-Fim2 after intranasal immunization was significantly higher.

### 3.5. Humoral Response in BAL Washes

**Figure 5:**
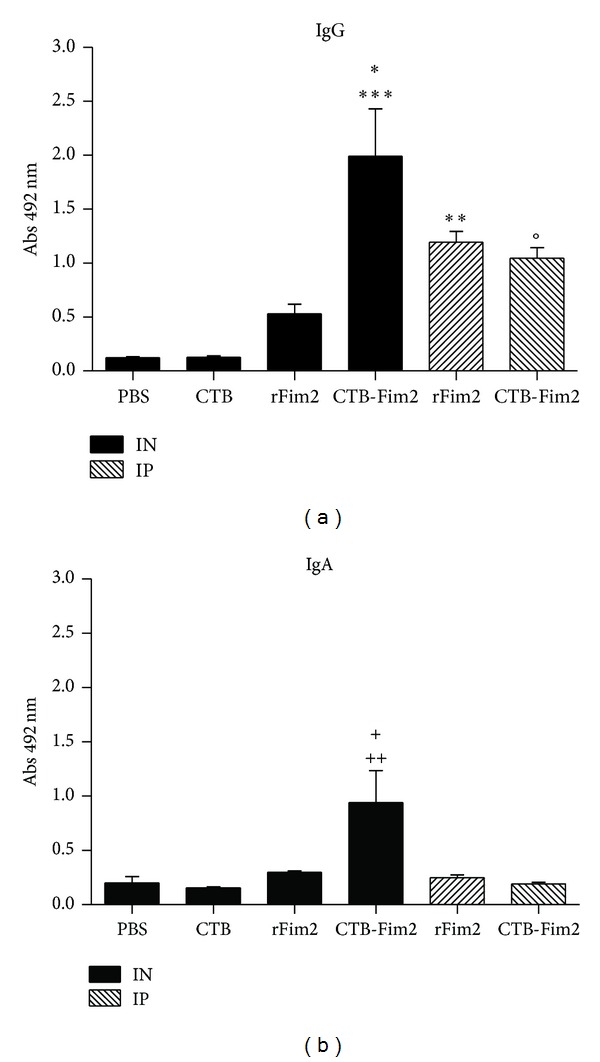
Specific IgG and IgA antibodies in BAL washes. Groups of animals were immunized intranasally or intraperitoneally three times with PBS, CTB, rFim2, or CTB-Fim2 every 12 days followed by intranasal challenge with* B. pertussis *Tohama phase I strain of (5 × 10^7^ CFU/mouse). At 8 days postinfection, animals were euthanized and BAL washes were collected to determine the levels of anti-Fim2 IgG (a) and IgA (b) antibodies. Antibody levels were assayed using ELISA. Values represent the OD at 492 nm and are representative of two separate experiments (*P < 0.05 versus CTB-Fim2 IP, **P < 0.001 and *P* < 0.0001 versus PBS, CTB, and rFim2 IN, °*P* < 0.05 versus PBS and CTB, ^+^ *P* < 0.05 versus PBS, CTB, and rFim2 IN and IP, and ^++^ *P* < 0.001 versus CTB-Fim2 IP).

To assess the immune responses at the mucosal surface, groups of mice were immunized intranasally or intraperitoneally three times with PBS, CTB, rFim2, or CTB-Fim2 every 12 days followed by intranasal challenge with* B. pertussis *Tohama phase I strain (5 × 10^7^ CFU/mouse). At 8 days postinfection animals were euthanized and BAL washes were collected for determination of anti-Fim2 IgG (a) and IgA (b) antibodies (Figure 5). Antibody levels were assayed using ELISA. Values represent the OD at 492 nm and are representative of two separate experiments (*P < 0.05 versus CTB-Fim2 IP, **P < 0.001 and ***P < 0.0001 versus PBS, CTB, and rFim2 IN, °*P* < 0.05 versus PBS and CTB, ^+^ *P* < 0.05 versus PBS, CTB, and rFim2 IN and IP, and ^++^ *P* < 0.001  versus CTB-Fim2 IP).

These results mainly indicate that rFim2, when administered intranasally, was not capable of generating a strong humoral response in BAL, but when CTB was genetically fused to the antigen, specific antibody levels significantly increased in BAL, giving high titers of IgG and IgA with the fusion protein.

### 3.6. Analysis of Th Cell Response

**Figure 6:**
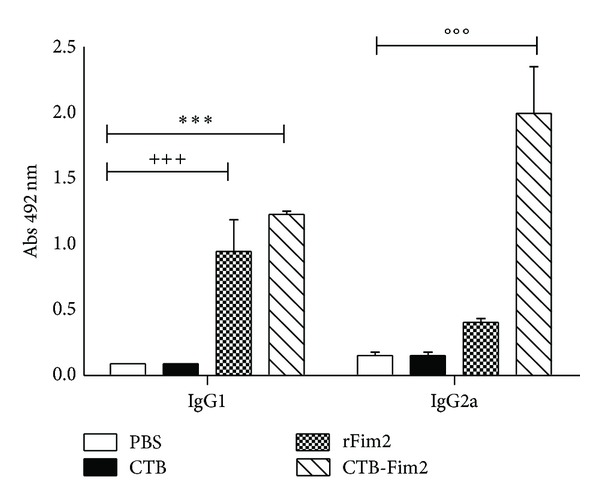
IgG1 and IgG2a antibody isotype response. Comparison of the serum anti-Fim2 IgG1 and IgG2a levels induced by PBS, CTB, rFim2, or CTB-Fim2 via the intranasal (IN) route. Sera were collected from mice on day 36. The anti-Fim2 IgG1 and IgG2a titers were determined using ELISA. Data are expressed as the mean (±standard error) absorbance at 492 nm for each group. Statistical differences were observed in different comparisons (IgG1 CTB-Fim2 versus all other categories except rFim2 ***P < 0.0001, rFim2 versus all other categories except CTB-Fim2 ^+++^ *P* < 0.0001, and IgG2a CTB-Fim2 versus all other categories °°°*P* < 0.0001).

**Figure 7:**
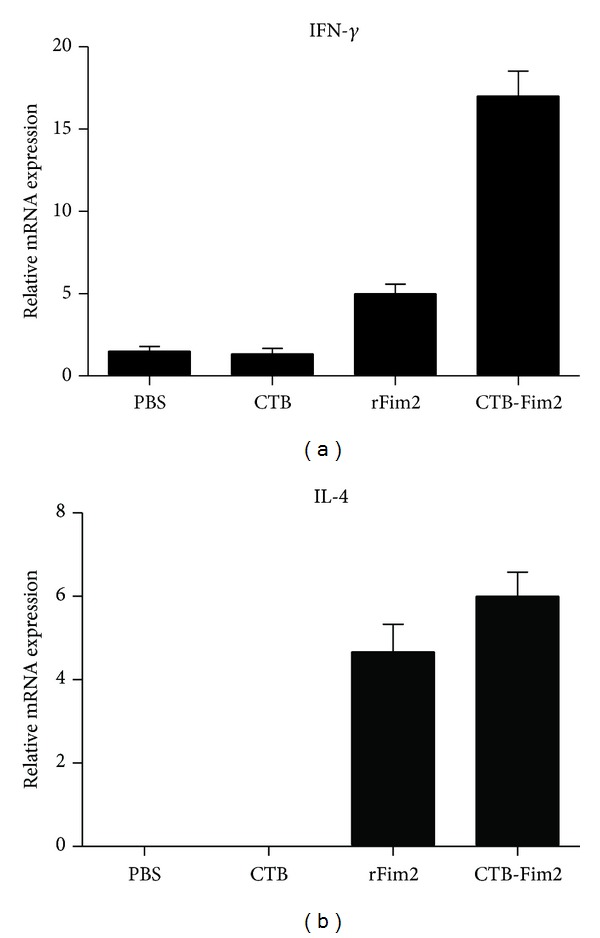
Cytokine mRNA expression levels in lung. Groups of animals were immunized intranasally three times with PBS, CTB, rFim2, or CTB-Fim2 every 12 days followed by intranasal challenge with 5 × 10^7^ UFC of the Tohama I strain of* B. pertussis*. At 8 days postinfection, animals were killed and lung samples were collected for measuring mRNA levels of IFN-*γ* (a) and IL-4 (b). Statistical differences were observed between CTB-Fim2 and all other groups except rFim2 (*P* < 0.001).

**Figure 8:**
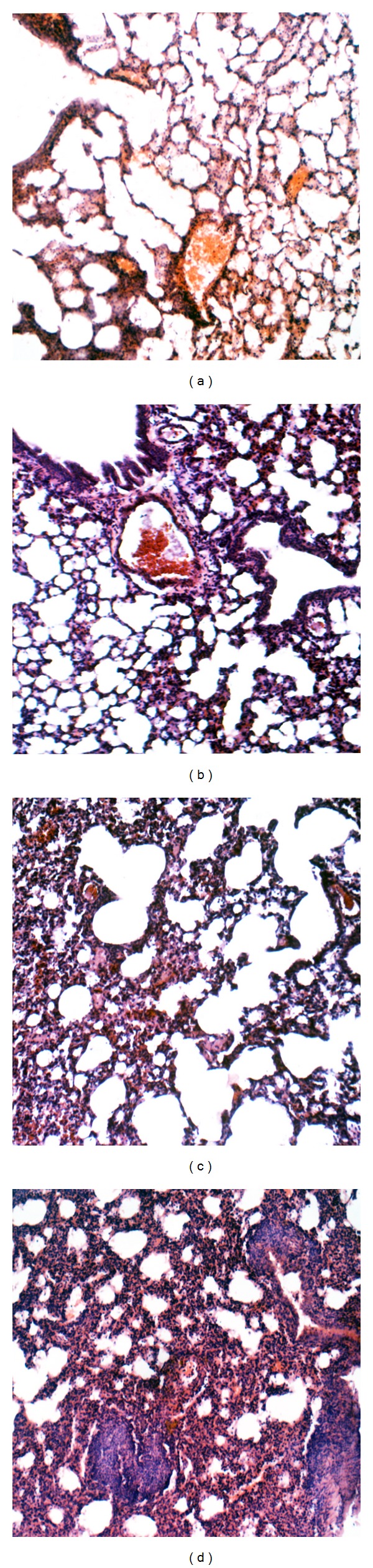
Representative lung sections of BALB/c mice after* B. pertussis *challenge. Mild lung inflammation in PBS (a) and CTB (b) immunized mice. Moderate lung inflammation in Fimb2 immunized animals (c). More severe lung inflammation with extensive cell exudate and some consolidation (pneumonia) was observed in lungs of CTB-Fimb2 immunized animals (d). Hematoxilin-eosin staining in all cases. ×150 magnifications.

To gain partial insight into the nature of immune responses induced by intranasal immunization with CTB-Fim2, we first quantified serum IgG subtypes G1 and G2a specific to Fim2 at day 36 postimmunization and before challenge (Figure 6). In agreement with specific total IgG response values found in serum, significantly higher levels of IgG1 antibodies were detected in the rFIM2 and CTB-Fim2 groups compared with the negative controls (DO at 492 nm = IgG1: 1.1 and 1.3, resp.; *P* < 0.05). In contrast, the IgG2a anti-Fim2 levels were only elevated by CTB-Fim2 (DO at 492 nm = IgG2a: 0.4 and 1.8, resp., *P* < 0.01). On the other hand, analysis of IFN-*γ* and IL-4 mRNA levels (Figure 7) showed that meanwhile rFim2 and CTB-Fim2 induce an increase of IL-4, only CTB-Fim2 induces a significant increase of IFN-*γ* in the lung of immunized mice compared with the negative controls. In addition, the histopathology analysis (Figure 8) showed a more intense inflammation in the lungs of mice immunized with CTB-Fim2 compared with the rFim2 and negative control groups. The enhanced inflammatory response detected at day 8 after the challenge was not deleterious for the animal evaluated as survival or presence of symptoms, including lethargy, hypothermia, or changes in nose/mucosal surfaces.

## 4. Discussion

Natural infection with* B. pertussis* induces strong and long-lasting immunity that wanes later than vaccine-induced immunity [33]. The use of the mucosal route of vaccination is an attractive alternative to the use of the parenteral route since it may mimic many aspects of the immune response elicited against the natural infection [34]. Moreover, a single nasal dose of a live attenuated* B. pertussis *was effective against whopping cough in the murine model [35]. In addition, other pertussis antigens such as pertactin and filamentous hemagglutinin or even the more complex component, the outer membrane vesicles administered via the mucosal route, has proven to be effective against* B. pertussis* infection [36, 37], showing that mucosal vaccination may constitute a possible alternative to the widely used parenteral route.

The presence of fimbriae (serotypes 2 and 3) in acellular pertussis vaccines has been shown to improve short-term vaccine efficacy in young children [38, 39]. In later studies, it was also described as a correlation between IgG anti-Fim2/3 and a reduced risk of disease [40, 41]. Although it has been well established that Fim2 purified from* B. pertussis *confers protection against infection in the mouse respiratory model [20], mice intraperitoneal immunized with the recombinant protein (rFim2) are not protected against challenge with *B. pertussis* [42]. The molecular basis of this difference has not been established, but it could be due to differences in the structure of Fim2 obtained from the two sources. Furthermore, the protective capacity of rFim2 delivered mucosally has not been reported previously.

Here we constructed CTB-Fim2 to explore Fim2 immunogenicity exploiting the adjuvant properties of the CTB molecule. CTB has been widely used as an adjuvant in rodent studies [13, 14]. When given via the oral or intranasal route, CTB not only elicits anti-CTB responses at multiple mucosal sites but also induces strong antibody responses to genetically fused administered antigens [32]. Previous studies have successfully tested the possibility of intranasal immunization with an acellular pertussis vaccine using CTB as an adjuvant, mixing recombinant CTB with pertussis toxoid and formalin-treated filamentous hemagglutinin [43]. Furthermore, a chimeric protein consisting of a divalent pertussis toxin (PTX) S1 fragment linked to the cholera toxin A2B fragment elicited protective immunity after three intranasal immunizations, showing the potent effect of CT as a mucosal adjuvant [44].

The CTB-Fim2 presented here formed a pentameric structure that seemed to be important in relation to its adjuvant capacity [13]. More importantly, a significant reduction in the number of bacteria recovered from the lungs was observed in mice immunized with CTB-Fim2 compared to those immunized with rFim2 or the control mice groups indicating that some antigen can offer protection when fused to CTB. The protective capacity of CTB-Fim2 over rFim2 may be due, at least in part, to both the increased uptake of coupled antigen across the mucosal barrier [45] and more efficient delivery to antigen-presenting cells [46]. In our conditions and in agreement with other authors [42], rFim2 alone was not a good immunogen to reduce bacterial burden in lungs when administered via either the intraperitoneal or the intranasal route.

We also observed that intranasal immunization with CTB-Fim2 induced similar levels of anti-Fim2 serum IgG to those of intraperitoneal immunization. Furthermore, intranasal immunization with CTB-Fim2 induced high levels of anti-Fim2 IgG in BAL, indicating that both CTB-Fim2 and intranasal administration of the immunogen are adequate to induce a strong IgG response in the lungs.

Since one of the hallmarks of mucosal immunity is the production of secretory IgA, which acts to prevent bacterial and viral infection, and since CTB is capable of inducing specific IgA [47–51] by synergism with MyD88-dependent TLR signals which selectively imprint a IgA-inducing capacity in nonmucosal DCs [52], we studied this immunoglobulin in CTB-Fim2-immunized mice and verified that mice administered CTB-Fim2 elicited specific Fim2-IgA in BAL. This result was not observed with the other treatments performed herein and is consistent with previously published data showing that parenteral administration is less effective than mucosal routes in inducing mucosal responses important for protection [53]. In transcutaneous and intranasal immunization with a* Chlamydia muridarum* antigen, higher levels of specific IgG were induced in serum and BAL, while only intranasal immunization induced specific IgA in BAL using CTB as an adjuvant [54]. It seems that CTB may directly cause B cells to enhance S-IgA production [55] and the IgA isotype switching through TGF-*β*1 [56].

In mice that were immunized by mucosal administration of CTB-Fim2, we observed a comparable rise of IgG1 and IL4 mRNA levels and enhanced IgG2a and IFN-*γ* mRNA levels as well as an increased inflammatory cell exudate. The enhanced inflammatory response in vaccinated mice, in agreement with other studies [57], may represent an enhanced host response to clear the bacteria. Taken together, the results showed that rFim2 results mainly in a Th2 response, as observed with aP vaccines [4], and that when rFim2 is fused to CTB, results in a mixed Th1/Th2 response, consistent with similar observations reported by others [58, 59] and improving one of the shortcomings observed in aP vaccines [60].

## 5. Conclusions

In conclusion, the results presented herein show that CTB is a potent adjuvant that when fused to Fim2 enhances their immunogenicity, stimulating both systemic and mucosal immune responses resulting in a mixed Th1/Th2 T-helper response. Having in mind the concerns that arose by the intranasal use of CTB and its subsequent accumulation in the olfactory bulb of mice [61] and that some vaccines administrated by the intranasal route have been associated with Bell's palsy in humans [62], the fact that CTB-Fim2 administrated by the intraperitoneal route also offers protection, supports its value as a protective immunogen. In addition, open the possibility to be used by other routes, even mucosal routes, as have been done with CTB fused to other antigens in preclinical studies [63] as well as in humans (CTB administered by mucosal way in healthy adult volunteers, trial NCT00820144, NIH, publication pending). Therefore, the genetic fusion of* ctb* and* fim2* genes provides a new promising antigen against* Bordetella pertussis *infection.
